# Papillary Serous Carcinoma of the Uterine Cervix with Lung Metastasis

**DOI:** 10.1155/2014/683103

**Published:** 2014-03-04

**Authors:** Maliha Khan, Alan D. Gilman, Sobia Nizami, Aram Barbaryan, Alaa M. Ali, Aibek E. Mirrakhimov

**Affiliations:** ^1^Department of Internal Medicine, Saint Joseph Hospital, 2900 North Lake Shore, Chicago, IL 60657, USA; ^2^Department of Medicine, Agha Khan University, Karachi, Sindh, Pakistan

## Abstract

Papillary serous carcinoma of the uterine cervix is a rare histological variant of cervical adenocarcinoma, with a very small number of cases reported. It is an aggressive tumor and is usually diagnosed at advanced stages by the time of diagnosis. Early-stage tumors can be treated with surgery and/or radiotherapy, while late-stage tumors have been treated with chemotherapy plus radical surgery with intermittent success. Here we report a case of metastatic papillary serous carcinoma observed at our hospital, which has been treated with debulking surgery and combination chemotherapy with carboplatin and paclitaxel.

## 1. Introduction

Cervical adenocarcinoma accounts for 10–20% of invasive cervical cancers and has a poor radiosensitivity and chemosensitivity [1, 2]. Papillary serous carcinoma of the uterine cervix (PSCC) is a very rare variant of cervical adenocarcinoma, which histologically resembles the same tumor occurring more commonly in the ovary, fallopian tube, endometrium, and peritoneum [3]. It is recognized as an aggressive neoplasm that can be pure or mixed with other adenocarcinoma subtypes [3]. PSCC is staged similarly to other types of cervical cancer and is presented in Table 1 [4]. It is usually found with lymph node metastases and occasionally in Stage III or IV (staging is presented in Table 1) [2]. The diagnosis of PSCC should be made after metastasis from other sites, particularly the endometrium, has been excluded [5]. There is a paucity of the literature on PSCC with only 46 cases being reported to date [2]. Here we describe a recent case of primary PSCC with pulmonary metastases diagnosed at our hospital.

## 2. Case Presentation

A 64-year-old African American female, gravida (G) 0 with menopause at age 52, presented with a four-week history of vaginal bleeding in July 2013. The bleeding occurred daily, ranging in severity from spotting to moderate bleeding. Her review of systems was otherwise negative, and she had no documented past medical history. She reported that her last Pap smear was performed several years ago and was normal. The vital signs were unremarkable. On pelvic examination, cervical ulceration of 2-3 cm with friability was found. The physical examination was otherwise unremarkable.

**Figure 1:**
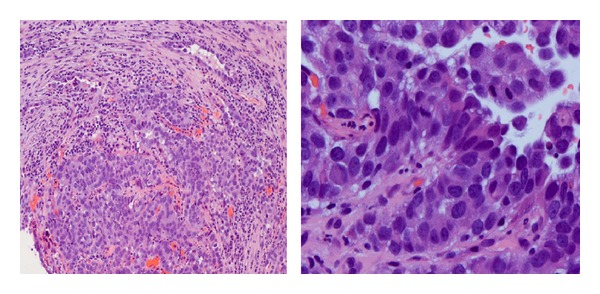
Cervical biopsy showing papillary serous carcinoma. The low power hematoxylin and eosin stain (4x) shows sheets of neoplastic cells embedded in fibrous tissue. The higher power hematoxylin and eosin stain (40x) shows the neoplasm having papillary architecture with hyperchromatic nuclei and marked nuclear pleomorphism.

**Figure 2:**
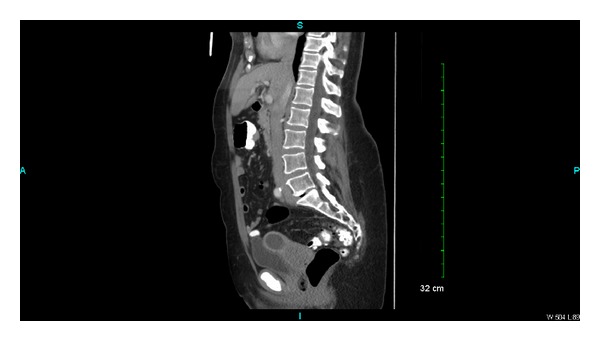
CT scan of abdomen and pelvis showing fluid-filled and distended uterine endometrial cavity with free fluid in the posterior cul-de-sac.

**Figure 3:**
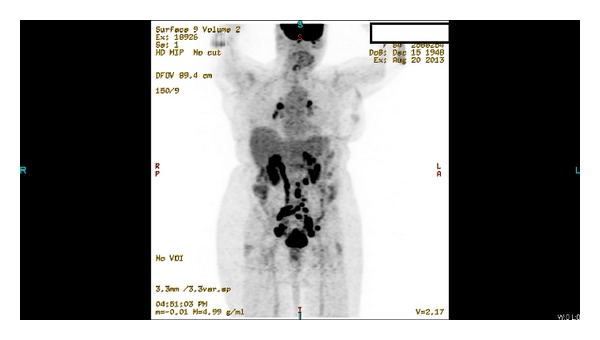
PET CT scan showing cervical mass, consistent with carcinoma, with diffuse lymphadenopathy throughout the abdomen, pelvis, and the hila of both lungs.

**Figure 4:**
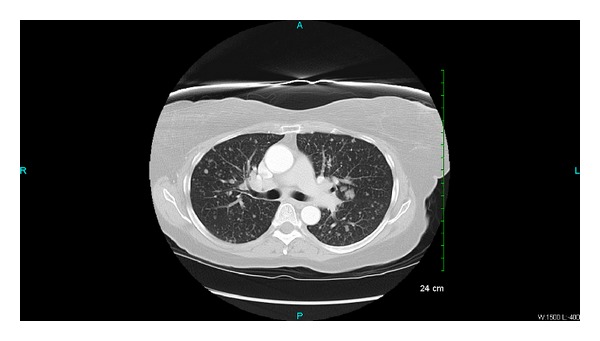
Chest CT scan showing multiple diffuse noncalcified nodules in both lung fields consistent with metastases.

**Figure 5:**
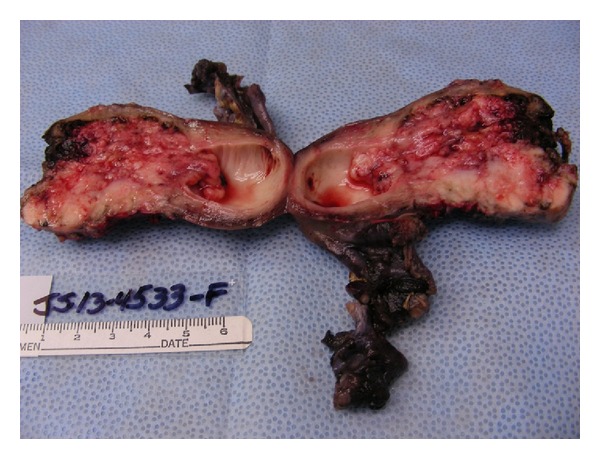
Hysterectomy specimen showing replacement of lower uterine segment and cervix with papillary serous carcinoma.

Laboratory investigations showed only microcytic anemia with a hemoglobin level of 11.3 g/dL (normal range: 14.0–18.0 gm/dL) and CA-125 of 343 (normal range: 0–35 U/mL). On transabdominal ultrasound, a small amount of fluid within the endometrial cavity was demonstrated, with no significant endometrial thickening. A cervical biopsy was performed, which revealed papillary serous carcinoma with mitotic activity at 4 mitotic figures per 10 high-power fields and occasional psammoma bodies (please see Figure 1). The immunostaining was positive for Ki-67 and p53 and negative for estrogen (ER) and progesterone (PR) receptors. A computed tomography (CT) scan of the abdomen and pelvis showed a fluid-filled and distended uterine endometrial cavity with free fluid in the posterior cul-de-sac (please see Figure 2). Positron emission tomography (PET) scan revealed extensive lymphadenopathy throughout the abdomen, pelvis, and bilateral hilar lung regions, along with multiple diffuse noncalcified nodules in both lung fields consistent with metastases identified on CT scan of the chest (please see Figures 3 and 4, resp.). Based on imaging and Ki-67 immunopositivity, the main tumor was determined to be located in the uterine cervix with no extension into the vaginal or uterine walls. It was staged as IVB, based on the revised FIGO staging for cervical cancer (please see Table 1). Given the tumor's advanced stage at presentation, she underwent debulking surgery with total abdominal hysterectomy and bilateral salpingo-oophorectomy in September 2013 (please see Figure 5), followed by combination chemotherapy with carboplatin and paclitaxel. The patient received four cycles of chemotherapy so far and tolerated it well without any major side effects.

## 3. Discussion

Adenocarcinomas of the uterine cervix represent approximately 10–20% of invasive cervical carcinomas, with an increasing incidence over recent years [6], and endocervical types account for approximately 70% of adenocarcinomas of the uterine cervix [1, 2]. PSCC is one of the rarely encountered and recently described subtypes of endocervical adenocarcinoma in the past 15 years [1, 7, 8]. Gilks and Clement first reported this entity in detail in 1992 and suggested an aggressive nature of this rare neoplasm [9]. To our knowledge, only 46 cases of PSCC have been reported in the literature and only one large series of 17 cases has been documented by Zhou et al. [2, 3]. Its pathogenesis is likely to be related to papillary serous carcinoma of the genital tract and peritoneum, since these tumors demonstrate similar microscopic features [5, 8]. As an aggressive neoplasm, it needs to be distinguished histologically from other papillary carcinomas of the cervix that are associated with a better prognosis, such as villoglandular papillary adenocarcinoma [5, 8]. Microscopically, the tumor appears in a papillary or glandular pattern, with tumor cells exhibiting hyperchromatic nuclei, with usually more than 10 mitotic figures and occasional psammoma bodies and these changes were also seen in our case [3].

A bimodal age distribution of PSCC has been noted, with one peak occurring before the age of 40 years and the second peak occurring after the age of 54 years [3, 8]. The patient presented in our case was diagnosed at age 52, stood a apart from the typical age distribution. The commonly noted presentations are abnormal vaginal bleeding or discharge, while some cancers are detected by a screening Pap smear in an asymptomatic patient [3, 10]. As observed in our patient, cervical examination can demonstrate ulcers or exophytic or polypoid masses [3]. The purpose of imaging is to not only investigate tumor spread and metastases but to also rule out a different primary tumor source with cervical metastasis [2]. In our case, no ovarian, peritoneal, or uterine masses were found; therefore, the cervical lesion was considered primary. The most common sites of metastases are pelvic and periaortic lymph nodes; other sites are reported as cervical lymph nodes, peritoneum, lung, liver, and skin [3]. In this case, the lung and lymph nodes were the sites of metastases. Poor prognosis has been associated with age of <65 years, stage >I, tumor size >2 cm, tumor invasion >10 mm, the presence of lymph node metastases, and elevation of serum CA-125 [3].

Immnunohistochemistry is helpful in diagnosing this rare entity [8]. Immunopositivity for p53, as observed in our case, marks an early event in tumor development as it was diffusely detected from PSCC in situ [11] and has been postulated to account for the aggressive behavior of PSCC [8, 12]. Higher p53 reactivity and lower carcinoembryonic antigen (CEA) reactivity are associated with a histological diagnosis of PSCC as compared to cervical adenocarcinomas of other subtypes [8].

As a relatively rare entity and recently described variant, optimal treatment of PSCC is still a matter of debate [13]. In the largest series of Zhou et al., 6 of 15 patients died of carcinoma, an outcome similar to that observed in adenocarcinoma of the cervix overall [3]. For stages I and II PSCC, suggested treatment strategies include surgery or radiotherapy alone [14] or primary surgical therapy followed by postoperative radiotherapy [9]. However, with an aggressive nature of the neoplasm, patients are usually diagnosed in stage III or IV with supradiaphragmatic metastasis and fatal outcomes especially in older patients [3, 8]. A lack of response of PSCC to chemotherapy with paclitaxel and carboplatin has been reported [3]. Recently, however, Ueda et al. reported an excellent response of stage IVb PSCC to primary combination neoadjuvant chemotherapy with paclitaxel and carboplatin prior to debulking surgery [2]. The plan for our patient is based on similar lines, with total abdominal hysterectomy and bilateral salpingo-oophorectomy to be followed by combination chemotherapy.

## 4. Take Home Points

- Papillary serous carcinoma of the uterine cervix (PSCC) is a rare histological variant of cervical adenocarcinoma.
- It is an aggressive tumor, reported to be poorly responsive to chemotherapy and radiotherapy.
- Most cases are diagnosed in advanced stages with metastases and require debulking surgery.
- Postoperative chemotherapy or radiotherapy is usually given; however, definitive recommendations on management are yet to be synthesized due to limited evidence.

## Figures and Tables

**Table 1:** Carcinoma of the cervix uteri: Féderation Internationale de Gynécologie et d'Obstétrique (FIGO) staging system (adapted from Reference [4]).

Stage	
I	The carcinoma is strictly confined to the cervix (extension to the corpus would be disregarded).
IA	Invasive carcinoma, which can be diagnosed only by microscopy with deepest invasion ≤5 mm and largest extension ≥7 mm.
IA1	Measured stromal invasion of ≤3.0 mm in depth and extension of ≤7.0 mm.
IA2	Measured stromal invasion of >3.0 mm and not >5.0 mm with an extension of not >7.0 mm.
IB	Clinically visible lesions limited to the cervix uteri or preclinical cancers greater than stage IA^b^.
IB1	Clinically visible lesion ≤4.0 cm in the greatest dimension.
IB2	Clinically visible lesion >4.0 cm in the greatest dimension.
II	Cervical carcinoma invades beyond the uterus but not to the pelvic wall or to the lower third of the vagina.
IIA	Without parametrial invasion.
IIA1	Clinically visible lesion ≤4.0 cm in the greatest dimension.
IIB2	Clinically visible lesion >4.0 cm in the greatest dimension.
IIB	With obvious parametrial invasion.
III	The tumor extends to the pelvic wall and/or involves lower third of the vagina and/or causes hydronephrosis or nonfunctioning kidney unless they are known to be due to other causes.
IIIA	Tumor involves lower third of the vagina with no extension to the pelvic wall.
IIIB	Extension to the pelvic wall and/or hydronephrosis or nonfunctioning kidney.
IV	The carcinoma has extended beyond the true pelvis or has involved (biopsy proven) the mucosa of the bladder or rectum. A bullous edema, as such, does not permit a case to be allotted to stage IV.
IVA	Spread of the growth to adjacent organs.
IVB	Spread to distant organs.

^b^The depth of invasion should not be more than 5 mm taken from the base of the epithelium, either surface of glandular epithelium, from which it originates.

## References

[1] Young RH, Scully RE (1990). Invasive adenocarcinoma and related tumors of the uterine cervix. *Seminars in Diagnostic Pathology*.

[2] Ueda M, Koshiyama M, Yamaguchi A (2012). Advanced papillary serous carcinoma of the uterine cervix: a case with a remarkable response to paclitaxel and carboplatin combination chemotherapy. *Rare Tumors*.

[3] Zhou C, Gilks CB, Hayes M, Clement PB (1998). Papillary serous carcinoma of the uterine cervix: a clinicopathologic study of 17 cases. *American Journal of Surgical Pathology*.

[4] Pecorelli S (2009). Revised FIGO staging for carcinoma of the vulva, cervix, and endometrium. *International Journal of Gynaecology and Obstetrics*.

[5] Young RH, Clement PB (2002). Endocervical adenocarcinoma and its variants: their morphology and differential diagnosis. *Histopathology*.

[6] Smith HO, Tiffany MF, Qualls CR, Key CR (2000). The rising incidence of adenocarcinoma relative to squamous cell carcinoma of the uterine cervix in the United States—a 24-year population-based study. *Gynecologic Oncology*.

[7] Shintaku M, Ueda H (1993). Serous papillary adenocarcinoma of the uterine cervix. *Histopathology*.

[8] Nofech-Mozes S, Rasty G, Ismiil N, Covens A, Khalifa MA (2006). Immunohistochemical characterization of endocervical papillary serous carcinoma. *International Journal of Gynecological Cancer*.

[9] Gilks CB, Clement PB (1992). Papillary serous adenocarcinoma of the uterine cervix: a report of three cases. *Modern Pathology*.

[10] Zhou C, Matisic JP, Clement PB (1997). Cytologic features of papillary serous adenocarcinoma of the uterine cervix. *Cancer*.

[11] Nofech-Mozes S, Khalifa MA (2009). Endocervical adenocarcinoma in situ, serous type. *International Journal of Gynecological Pathology*.

[12] Gerard Power D, Paul McVey G, William Delaney D (2008). Papillary serous carcinomas of the uterine cervix and paraneoplastic cerebellar degeneration: a report of two cases. *Acta Oncologica*.

[13] Geisler JP, Hiett AK, Geisler HE, Shade A, Cudahy TJ, Moore DK (1998). Papillary serous carcinoma of the cervix: ultrasonographic findings. *European Journal of Gynaecological Oncology*.

[14] Rose PG, Reale FR (1993). Serous papillary carcinoma of the cervix. *Gynecologic Oncology*.

